# The ‘silent’ half: diversity, function and the critical knowledge gap on female frog vocalizations

**DOI:** 10.1098/rspb.2025.0454

**Published:** 2025-05-28

**Authors:** Erika M. Santana, Angela M. Mendoza-Henao, Johana Goyes Vallejos

**Affiliations:** ^1^ Instituto de Biociências, Universidade de São Paulo, São Paulo, Brazil; ^2^ Red Ecoacústica Colombia, Cali, Colombia; ^3^ Division of Biological Sciences, University of Missouri, Columbia, MO, USA

**Keywords:** Amphibia, Anura, animal communication, female anuran call, female strategies, sex-specific calls

## Abstract

Anuran vocalizations are crucial for species recognition and social interactions, particularly in reproduction. Historically, research has focused almost exclusively on male calls, leading to a male-biased perspective in anuran bioacoustics. Female calls have been often neglected due to their softness, making them difficult to detect. This review provides an overview of female calling behaviour in anurans, addressing a critical gap in frog bioacoustics and sexual selection. Specifically, we aim to (i) provide an overview of the current state of knowledge of female calling in frogs, (ii) propose a standardized classification for anuran call types, (iii) identify general patterns and challenges, (iv) recommend best practices, and (v) highlight areas for further exploration. Our literature review indicates that female calls have been documented in 112 species across 53 genera and 29 families, representing approximately 1.43% of all described anuran species. However, most descriptions are anecdotal or purely descriptive, with few functional analyses. Our findings underscore the widespread but largely overlooked presence of calling females in anurans. Expanding our research efforts on female vocalizations will improve our understanding of anuran communication. We hope this review motivates researchers to consider female frogs in future behavioural, ecological and evolutionary studies.

## 1. Introduction

Animal vocal communication plays a vital role in interspecific and intraspecific interactions related to survival, resource acquisition and reproduction [1]. In many taxa, acoustic communication is essential for mating competition and choice, territorial defence, and signalling during combat, providing fitness-related information to signallers [2]. In addition, acoustic signals may serve important social functions, such as alarm calls that alert the group to potential threats and detecting and conveying information about food availability [3]. Given the importance of acoustic communication for a species’s fitness, a solid empirical understanding of the contexts in which acoustic signals are produced is crucial to explaining why animals vocalize and how this behaviour evolved.

Despite the prevalence of animal taxa that produce sounds, research on acoustic communication has historically focused on male signals [4]. This bias stems from the expectation that males are those who participate in aggressive competition and intersexual displays, including loud agonistic encounters or elaborate singing repertoires [2]. Consequently, behavioural, ecological and bioacoustics research has predominantly focused on describing, analysing and understanding the variation and function of male acoustic signals, often with little or no regard for their female counterparts [5–7]. A prominent example of this bias is evident in the history of birdsong studies. Until recently, research focused almost exclusively on male birdsong, often attributing female birdsong to a by-product of selection on male traits or dismissing it as a rarity among females [8]. However, current studies have demonstrated that female song is not only ancestral to modern songbirds but also widespread, with two-thirds of songbird species featuring female vocalizations [7,9]. Signalling females are not only present in birds [10] but also in mammals [11,12], reptiles [13–15] and many insect species [16]. Across taxa, the historical overlooking of female vocalizations and their roles has hindered our understanding of species’ intra and interspecific interactions and, consequently, the selective pressures to which they are subjected.

In frogs, call production plays a crucial role in reproductive behaviour and species recognition. The vocal repertoire of each species is predominantly produced by males, which has led to a focus on anuran male calls in bioacoustics research [17]. Male calls serve multiple functions, including attracting females, stimulating hormone production to maintain reproductive status, advertising a male’s position to conspecific males and enabling individual recognition of neighbours (reviewed in [17]). Conversely, it has been assumed that female frogs remain silent and, for decades, reports of female frog vocalizations (hereafter ‘female calls’) were overlooked or dismissed. This assumption is based on the absence of vocal sacs in females, which frequently makes their calls soft and difficult to detect. However, female frogs exhibit a rich repertoire of behaviours for interindividual communication, including visual signalling [18], body vibrations [19] and various forms of acoustic communication [20–22]. Moreover, female vocalizations in anurans have been documented as early as 1906 [23], and reports on female calling have steadily increased over the years. Despite this, female vocalizations remain poorly understood, since most studies to date have focused solely on reporting their occurrence. Thus, there is a pressing need for research that goes beyond merely documenting isolated instances of female calls—such as exploring the functions, mechanisms, behavioural and ecological roles and evolutionary patterns of these vocalizations—shedding light on a neglected aspect of amphibian communication.

In this review, we examine the literature on female calls to assess the current state of knowledge on this topic across the fields of bioacoustics, animal communication, behavioural ecology and herpetology. First, we provide an overview of female calls, including their historical classification and the need for clarity and consensus in definitions. Second, we propose a standardized classification for anuran call types (both male and female). Third, we provide a comprehensive account of how many anuran species are known to produce female calls, offering new insights into the extent and distribution of this behaviour within anurans—a scope of research that has not been systematically explored in decades. Finally, we identify general patterns, disparities and gaps in the existing research, outlining the main challenges in studying female calls and emphasizing key areas for further investigation. We hope this review will inspire researchers to focus on female behaviours and strategies in future studies on anuran behaviour, ecology and evolution.

## 2. The anuran female calling repertoire

### (a) Historical overview

**Figure 1.**
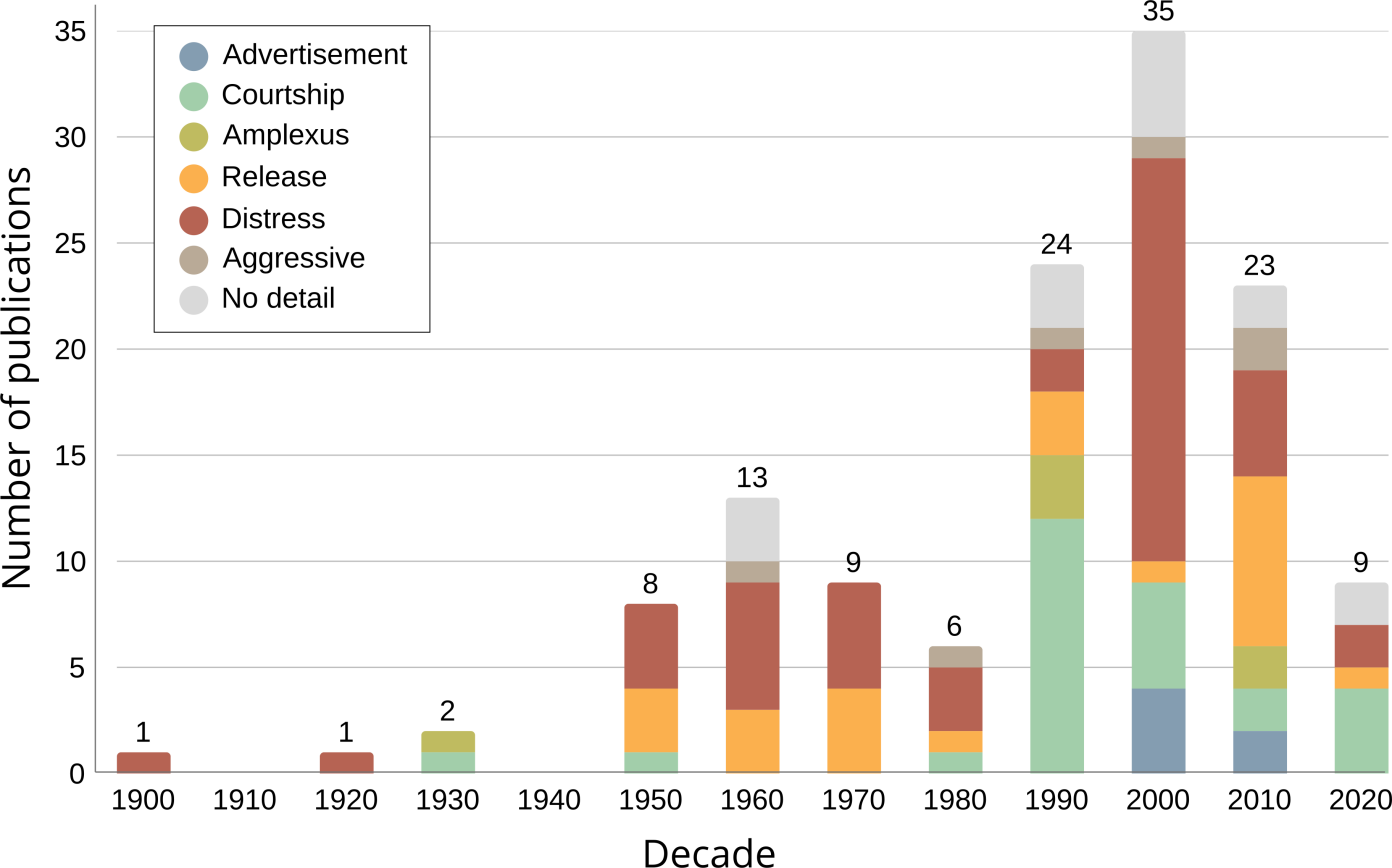
Trends in the publication history of anuran female calls, organized by the decade of publication and by call type (refer to §2c for definitions). The numbers above each bar represent the total number of publications for each decade.

The first documentation of female calls was in Mary C. Dickerson’s *The Frog Book* in the early 20th century [23]. Dickerson discussed how *Aquarana* (previously *Rana*) *catesbeiana* can produce the typical ‘jug-a-rum’ vocalizations as loudly as males. She also noted that in *Dryophytes* (previously *Hyla) arenicolor*, both males and females emit a high-pitched vocalization when handled. Given the limited context and information provided, we can only infer that the former could be a spontaneously produced courtship-type of call, while the latter is a distress call. Strikingly, despite these early observations of female calls, even in well-studied species like *A. catesbeiana* (see [24–27]), female calls are seldom mentioned in further studies. Dickerson [23] suggested that, while females may produce quieter vocalizations, further investigation might reveal that most females are capable of vocalizing in some form. This view, however, did not gain traction, as it later became widely assumed that only male frogs vocalize. Consequently, despite an increase in reports of female calls over the years (figure 1), the depth of these studies has not progressed significantly.

Since Dickerson’s work [23], reports of female calls were sparse until the late 1920s [28]. From then until the 1960s, only a few studies documented female calls, mostly in response to handling or disturbance. In 1932, Savage reported *Bombina variegata* females vocalizing when ready to mate, marking an early instance of reproductive rather than distress-related calls [29]. Similarly, in 1957, females of *Eleutherodactylus angustidigitorium* and *E. nitidus* were reported to vocalize in a context unrelated to human manipulation. In this study, details included the lack of vocal sacs in females, how the female calls were distinct from those of males and that these calls were likely produced in a courtship context [30]. From the 1950s onwards, reports of female calls increased, though most instances documented distress or release calls. However, few studies have gone beyond descriptive accounts to provide detailed observations of the context and role in which female calls are elicited.

### (b) The problem with definitions

The study of female calls faces an initial challenge: the lack of consistent and clear definitions. Researchers use a variety of terms and classifications, that range from vague mentions of female calls without context, to terms such as ‘breeding call’ [31], ‘close-range encounter call’ [32], ‘rejection signal’ [33], ‘duetting call’ [34], ‘stimulating call’ [35], ‘reciprocal call’ [20], ‘female sex call’ [36] and ‘mating-like call’ [37]. Moreover, most female call definitions are indirectly derived from frameworks established for male calls [21,38–41]. Significant discrepancies remain in how calls are defined, which has obstructed the synthesis of existing information on female vocalizations.

One of the earliest efforts to generally categorize anuran vocalizations was made by Bogert [19], who identified at least six call types based on the contexts in which they are produced: (i) mating calls, produced by sexually mature adults; (ii) territorial calls, used to respond to conspecific ‘intruders’ or maintain territory boundaries; (iii) male release calls and (iv) female release calls, emitted when individuals are not receptive or grasped by unwanted mates; (v) distress calls, produced when seized by potential predators, invariably produced with the mouth open; and (vi) warning calls, given during movements such as diving into water to evade predators. Bogert’s contribution was groundbreaking, not only for his effort to classify anuran calls within behavioural contexts, but also for his inclusion of male and female calls, exemplified by his detailed discussion of male and female release calls as distinct categories. He also recognizes the potential for certain call types (i.e. mating, distress and warning calls) to occur in both sexes. This ambisexual perspective was remarkably ahead of its time, as later research predominantly focused on male vocalizations.

In 1977, two consecutive studies provided greater detail on frog calls’ functional and contextual diversity. Littlejohn [42] introduced a signal transmission framework between sender and receiver, identifying four call types, one of which was Bogert’s territorial call *sensu stricto* [19]. The others included the ‘encounter call’, ‘reciprocal call’ and ‘advertisement call’, a term coined by Wells [43] to describe vocalizations with two distinct functions: (i) signalling territory occupation to both conspecific and heterospecific individuals, and (ii) attracting conspecific females, aligning with Bogert’s earlier concept of a mating call. Notably, reciprocal calls were the only call type explicitly attributed to females.

In his seminal work, Wells [43] made a groundbreaking contribution by highlighting the dual function of advertisement calls: attracting mates and signalling to other males about territory, position or body size during agonistic encounters. Later, in his comprehensive book *The Ecology and Behavior of Amphibians* [17], he provided an extensive review of call diversity and synthesized the current knowledge on vocal communication in anurans. Building heavily on Bogert and Littlejohn’s contributions, Wells categorized six primary call types and examined their contexts and functions [17]: advertisement calls, male courtship calls, aggressive calls, distress calls, release calls and female courtship calls (the only call type addressing female vocal behaviour). Wells argued that ‘aggressive’ is a more appropriate term than ‘territorial’, as some species produce aggressive calls without being territorial [17]. He also noted that encounter calls are essentially close-range aggressive calls and fall within the broader category of aggressive calls. Distress calls encompass vocalizations such as distress, alarm, defensive or warning calls, typically produced in response to predation attempts or when startled. Wells also emphasized that both sexes can produce release calls and suggested that these calls may be more widespread than currently recognized. Wells devoted a section of his review to this topic, but only briefly discussed female vocalizations, focusing predominantly on male calls throughout the chapter [17]. He described that female courtship calls are short-range vocalizations typically as responses to male advertisement calls, aligning with Littlejohn’s definition of female reciprocal calls [42].

A study by Toledo and collaborators [44] stands out as one of the first to highlight that both males and females produce vocalizations in nearly half of the proposed call types, not just distress calls. They also identified the amplexus call (a vocalization emitted during amplexus) as being produced by both sexes. However, existing definitions often shoehorn female calls into frameworks developed for male calls, without considering context, sex-specific functions or characteristics. Thus, we argue that definitions of anuran calls require refinement to align with our current knowledge. Our intention is to refine current definitions, rather than creating more definitions or overcomplicating existing ones.

### (c) Refining call definitions in anurans

**Figure 2.**
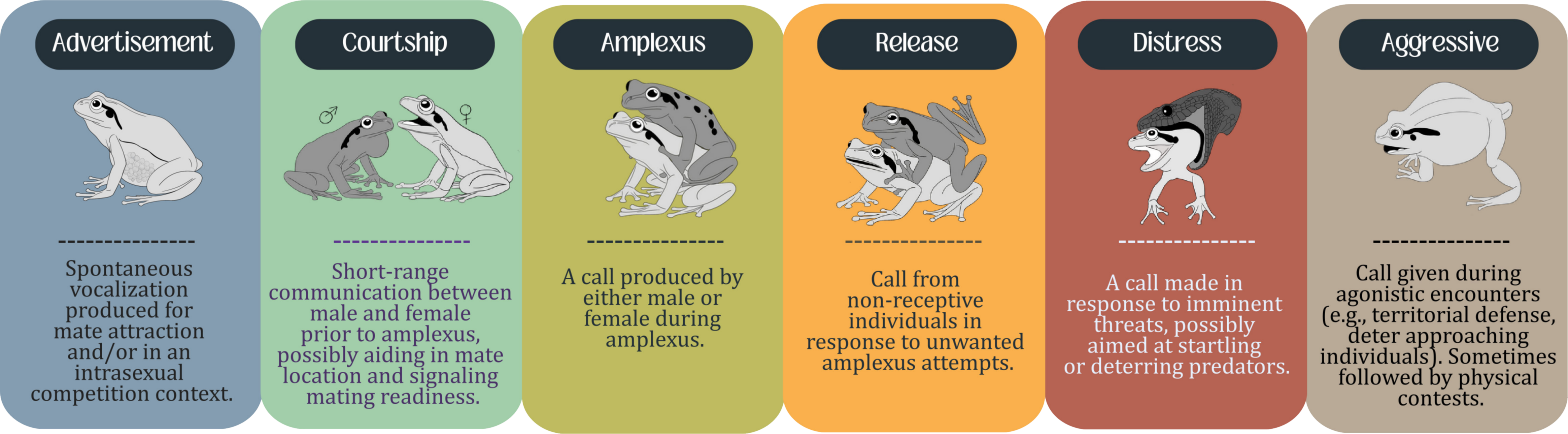
Classification of call types emitted by frogs. Females are represented in light grey, while males are represented in dark grey.

Over the last two decades, researchers have attempted to establish additional definitions for the variety of calls emitted by male and female anurans. However, these new definitions frequently end up as derivatives of previous ones, providing limited additional insights compared with the existing definitions and creating discrepancies in the literature. For a more standardized framework that allows future comparisons across studies and species, we propose six calling types (figure 2). These call types are applicable to both sexes and are defined as follows:

(i) *Advertisement call*. A spontaneous vocalization produced for mate attraction and/or in an intrasexual competition context linked to sexual selection processes (*sensu* [43,44]).
(ii) *Courtship call*. A close-range communication between a male and a female, occurring prior to amplexus, with suggested functions including stimulating calling activity, aiding in mate location and signalling readiness to mate. In some studies, this call type has been referred to as a ‘reciprocal call’*,* as the female’s call is typically elicited only after hearing an initial male advertisement call.
(iii) *Amplexus call*. A call produced while a male and female are engaged in amplexus. We know very little about the function of amplexus calls in both males and females.
(iv) *Release call*. A vocalization emitted by non-receptive individuals in response to an amplexus attempt by conspecifics (or, in some cases, heterospecifics) or undesired tactile stimulation associated with mating attempts. Both sexes may produce release calls, which occur exclusively in response to physical contact.
(v) *Distress call*. A generally open-mouth vocalization emitted in response to an immediate threat, such as a predator, typically when an individual is physically restrained or captured. The term encompasses defensive, alarm and warning calls as described in [17,41,44]. Adults defending their offspring may also produce distress calls, as observed in *Leptodactylus latrans* females [45].
(vi) *Aggressive call*. A vocalization produced during conspecific agonistic interactions, including territorial defence, typically in response to an approaching individual. These calls are presumed to serve as a deterrent before the conflict escalates. Aggressive calls may sometimes be followed by physical contests (fighting).

## 3. Female calling behaviour across anurans

### (a) Literature review

To identify studies on female calling behavior in amphibians, we conducted a systematic literature review, beginning in July 2023. We included peer-reviewed documents, journal articles, theses and conference abstracts (excluding preprints). Our search strategy included initial broad exploratory searches with subsequent targeted searches to maximize coverage. We used two primary databases: Google Scholar and Web of Science (WOS) with ‘Anura’ or ‘amphibian’ AND ‘calling female’ or ‘female acoustic response’ or ‘female vocal interaction’ or ‘female mating vocalization’ as key terms. We also applied subject area and taxonomic exclusionary filters to refine our results. We supplemented these results by conducting backward and forward citation searches in Google Scholar using three key review papers on female anuran vocalizations [40,46,47]. Combining these results with those from the initial Google Scholar and WOS searches yielded 2919 documents (detailed methods in electronic supplementary material, file S1).

We screened the titles and abstracts of all retrieved documents to assess relevance, reading in full those that explicitly mentioned female calls or focused on amphibian ecology, behaviour, physiology or bioacoustics. Sources were included if they had direct observations, spectrograms or detailed descriptions of female vocalizations. We also included sources in French, German, Portuguese and Spanish. This yielded 90 records. For relevant cases within this set, we conducted additional backward and forward citation searches adding 18 studies not captured by the initial search, resulting in 108 documents in total. Data were compiled in a standardized spreadsheet with taxonomic names checked and duplicates removed (electronic supplementary material, file S2). The search concluded in November 2024.

### (b) Data extraction and family-level mapping

We compiled the data on female calls from publications that specifically reported females calling. For each study, we extracted the species name, the type of call as defined by the authors, female call description (if available), and any ecological or behavioural contextual information about the vocalization. We also recorded whether the authors conducted experiments or behavioural assays to test the function of the female call. Then, we compared the female call definitions from the original studies to our proposed definitions. Whenever our classification differed from the reported one, we emphasized that our assignments relied solely on the authors’ information, without making any inferences beyond what was explicitly stated.

To standardize the data, we coded instances of spontaneous calling, researcher manipulation (i.e. handling or physical restraint), and tests of functional significance as binary variables (yes = 1, no = 0). When information was unavailable, we designated it as NA (not available). All extracted information was organized and summarized in a table (electronic supplementary material, file S2). We mapped the different types of female calls onto a consensus amphibian phylogenetic tree [48], which includes 54 extant anuran families. To account for cases where female calling was reported but lacked sufficient detail to classify the call type, we included a ‘no detail’ category.

**Figure 3.**
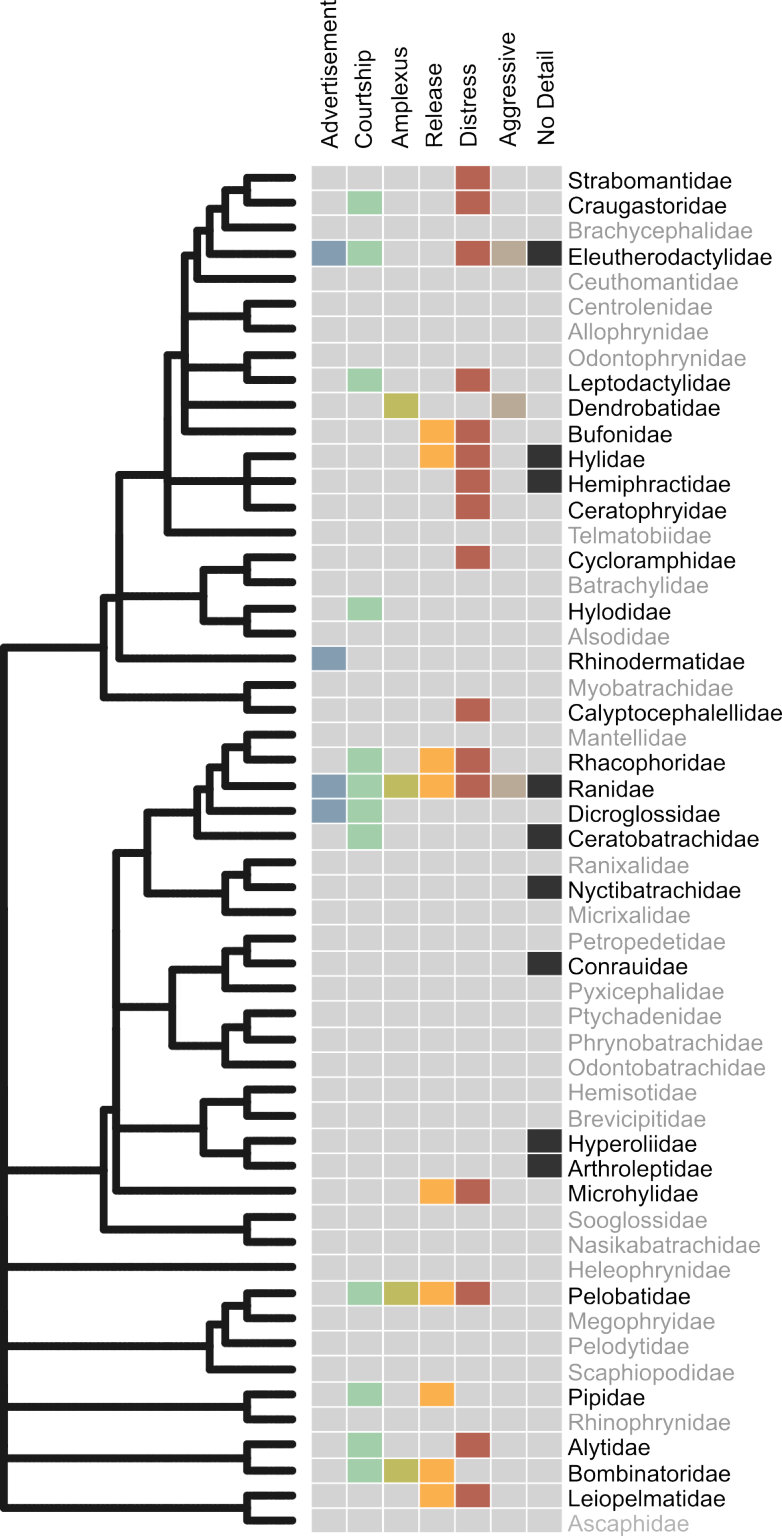
Distribution of female call records across anuran families mapped onto a phylogenetic tree. The heatmap illustrates the presence (coloured by call type) or absence (grey) of documented female calls. Call types follow the categories in figure 2. ‘No detail’ refers to calls with insufficient information for classification.

We recorded 132 instances of female calling behaviour across 112 species (53 genera, 29 families) from all the studies examined for this review. This represents approximately 1.43% of all the described anuran species. Female calling behaviour in frogs is widespread across the phylogeny (figure 3), occurring in both deeply divergent families like Bombinatoridae and Leiopelmatidae, as well as in more recently diverged families such as Craugastoridae and Rhacophoridae. Ranidae has the greatest number of species (18 species), followed by Hylidae (15 species), Leptodactylidae (14 species) and Eleutherodactylidae (10 species). At the genus level, *Leptodactylus* has the highest number of species with female calls with 12 species reported to produce female calls, followed by *Eleutherodactylus* with 10 species and *Xenopus* with eight species. We want to emphasize that the lack of records of female calls in certain families or genera does not necessarily indicate the absence of this behaviour. Instead, it reflects the insufficient knowledge of basic natural history and the lack of efforts in investigating female calling behaviour in frogs.

## 4. General patterns of female anuran calls

Female calls are phylogenetically widespread, occurring in over 50% of anuran families. Notably, the number of species with documented female calls has increased more than threefold compared with the last reviews on the subject [40,47]. The most frequently reported call types were distress (45 species), courtship (27 species) and release calls (24 species). Distress calls are documented in half of the frog families, indicating they are the most common call type across the clade. Advertisement and aggressive calls were each reported in six species, while amplexus calls were reported in five species. In 15 cases (11.4%), female calls were mentioned, but the authors did not provide sufficient details to classify them according to our proposed call types. We found agreement with the original authors’ definitions in 80 cases (60.6%). In 38 cases (28.8%), we assigned a new classification due to either a lack of explicit definitions, requiring us to classify the call based on contextual information found in the paper, or because the terminology was outdated or inconsistent with the behavioural context. Furthermore, in 89.6% (43 out of 48) reports of distress calls, the calls were elicited by researcher manipulation, including handling, restraint, hormone induction and more brutal methods like shaking the plastic bags or smacking the frog’s head (electronic supplementary material, file S2).

Although the number of species with female calling behaviour still constitutes only about 1% of all extant frog species, it is interesting to note that one-third of these vocalizations are linked to mate acquisition processes (i.e. advertisement, courtship and amplexus calls). This challenges the common perception of females as ‘silent choosers’, emphasizing their potential role as the competing sex and the possibility of mutual mate choice in species where male and female calls are used in courtship. These findings highlight a gap in our understanding of how sexual selection influences female calls and how it may act on both sexes when both vocalize.

Perhaps our most critical finding is that functional tests of female calls are virtually non-existent. Only 10% of the reported instances of female calls had some experimental assessment of function, such as behavioural experiments or playback tests (electronic supplementary material, file S2). This lack of functional tests, along with widespread misconceptions about female calling behaviour, stresses the need for more rigorous experimental approaches to elucidate the ecological and evolutionary roles of female calling in frogs (see §5c).

### (a) Distress and release calls

Distress and release calls in anurans have traditionally been studied with a male-biased perspective, despite early recognition that both males and females vocalize [49]. These calls occur in non-mating contexts, such as predator threats or unwanted amplexus attempts, which both sexes can experience. However, research on the evolution of distress calls has primarily focused on males, neglecting the possibility that these calls serve similar functions in both sexes [50,51]. We argue that both distress and release calls probably occur in both sexes and serve a species-level function rather than being sex-specific.

In female frogs, distress and release calls are the most commonly documented, yet their functions and evolutionary origins remain ambiguous. Most studies suggest distress calls serve as predator deterrents, though no experimental tests have directly tested this. Similarly, release calls have only been inferred from mechanical stimulation, such as grasping females to elicit a response. A well-executed study exploring the function of release calls was done in *Lithobates pipiens* [52]. Males and non-receptive *L. pipiens* females (non-gravid) produce release calls when clasped by males. In contrast, gravid females and females injected with a saline solution to mimic a gravid state do not produce a release call, suggesting that reproductive status influences females’ (but not males’) capability of producing release calls. Another example that incorporated a sex-inclusive perspective was done with two *Xenopus* species (*X. borealis* and *X. poweri*). Researchers tested whether male and female release calls were equally effective in ending unwanted amplexus and found that female release calls were less efficient at inducing release [21]. Such experimental approaches exploring female calls, alone or in conjunction with male calls, remain scarce yet are profoundly needed to move the field forward.

### (b) Courtship and amplexus calls

Female courtship and amplexus calls are emitted during close-range mating interactions. In this particular context, the former is the most frequently reported female call type, while the latter is among the least common. Courtship calls have always been relatively easy to identify (they typically follow a male advertisement call) and their classification as ‘courtship’ has never been debated. Likewise, amplexus calls occur exclusively when a male clasps a receptive female, leaving no doubt regarding their association with mating processes. While numerous hypotheses explain why females produce these calls [53–55], their reproductive role is often assumed rather than tested. The function in mating and the fitness benefits for females are still unclear, with only one species having undergone experimental testing for courtship call function [54,56]. Questions remain about whether courtship calls affect mate selection or whether amplexus calls influence reproductive success. For instance, in *Nidirana dauchina*, females call between the male’s rhythmic movements during amplexus. When researchers manually interrupted the male’s movement, the female continued calling until he resumed [35]. This suggests that amplexus calls may stimulate male mating behaviour, but their impact on fertilization or reproductive success remains unknown.

### (c) Spontaneous female calling in frogs

An interesting pattern identified from our data extraction is the occurrence of spontaneous female calling. We found 22 cases of spontaneous vocalizations, defined as vocalizations produced without tactile or acoustic stimuli (e.g. calling conspecific, attempted amplexus or human handling). These include female advertisement calls (see §4d), and aggressive calls, where females call aggressively in response to the approach of a conspecific (male or female), as seen in *E. coqui*, *Pelophylax porosus* and *Staurois guttatus* [33,40,57]. A few cases of spontaneous female calling have been observed in captivity, particularly when females were heavily gravid [36,58–61]. These vocalizations are likely to be courtship calls, emitted once they reach a threshold of reproductive readiness, possibly triggered by the risk of losing their clutch. Spontaneous calling has also been documented under manipulation, such as being separated from the male while in amplexus or after being placed in plastic bags or cages [30,46,58,62,63]. However, since all these observations were made in captivity or under artificial conditions, it is unclear whether females of these species call spontaneously in the wild. Overall, while spontaneous calling does occur, we suggest that, in a reproductive context, the most observed pattern is for females to emit courtship calls in response to male vocalizations.

A particular case of spontaneous vocalization not related to reproductive behaviours occurs in *Aquarana catesbeiana* [64] and *Pelophylax sculentus* [65], where females produce distress calls as they dive into the water, when startled. Despite these exceptions, we suggest that distress calls are not normally produced spontaneously.

### (d) The (female) advertisement call

Male advertisement calls are species-specific signals used in reproductive contexts and in species recognition, for mate attraction and signalling to conspecific males [17]. These calls have been widely used in taxonomy, functional tests and for examining evolutionary patterns of signal variation in anurans [41,66,67]. Traditionally, advertisement calls are attributed exclusively to males. In contrast, female vocalizations in an intraspecific context are designated as courtship calls. For too long, it has been assumed that the absence of vocal sacs in females impedes the production of advertisement calls. This raises the question: (i) can female frogs also produce advertisement calls? And if so, (ii) what distinguishes an advertisement call from a courtship call?

Wells [43] identified the dual function of advertisement calls since the intended receivers of these calls can be either potential mates or same-sex competitors. Courtship calls, on the other hand, are directed at specific individuals, invariably as a response to an initial acoustic stimulus (e.g. male advertisement calls). A prime example of the dual function of advertisement calls can be found in the coqui frog (*Eleutherodactylus coqui*). The male advertisement call of *E. coqui* consists of two distinct notes: the ‘co’ note and the ‘qui’ note. The ‘co’ note prompts male aggressive responses but the ‘qui’ note does not. Conversely, females are attracted to the ‘qui’ note, but they do not respond to the ‘co’ note alone [68]. Despite this evidence, most research on the function of male advertisement calls has focused on their role in attracting females (e.g. phonotaxis experiments), with less attention given to their potential to deter rivals.

We propose a new, refined definition of frog advertisement calls for both males and females. This definition accounts for cases where the primary intended receiver is a potential mate but also recognizes that same-sex individuals can be legitimate targets of these calls. Upon carefully evaluating the information extracted from the literature (i.e. authors’ call type definition, behavioural and ecological context), we compared it to our refined definition to determine in which cases female calls qualified as an advertisement call. Based on our definition, we documented six instances of female advertisement calls and dedicated this section to exploring their unique characteristics due to their rarity and significance.

In the American bullfrog *A. catesbeiana*, males and females produce vocalizations, with female calls being similar to male advertisement calls. Judge *et al*. [69] reported male–male aggressive interactions after a female was heard vocalizing. However, it remains unclear whether the female call directly triggered this behaviour. Breeding aggregations in this species are heavily male-biased, and females are encountered rarely, possibly explaining the lack of follow-up studies investigating female vocalization in bullfrogs.

Similarly, female Mexican chirping frogs *Eleutherodactylus cystignathoides* [62] produce spontaneous calls indistinguishable from the male calls and do not direct their calls towards any specific individual. This behaviour results in individuals orienting towards both male and female calls. This call monomorphism suggests that male and female *E. cystignathoides* calls serve as advertisement calls.

In Darwin’s frog (*Rhinoderma darwinii*), males and females produce monomorphic and indistinguishable advertisement calls during the breeding season, with similar spectral and temporal characteristics [70]. However, little is known about their courtship behaviour, how mate attraction is achieved or whether males attract females or vice versa. Thus, while the exact function of the female calls in *R. darwinii* remains unclear, they are probably involved in mate attraction. As a result, we concur with the authors’ classification of these vocalizations as advertisement calls.

Sexual monomorphism in calls is not a prerequisite for categorization as advertisement calls. In the bicoloured forest frog *Clinotarsus curtipes*, an endemic species from the Western Ghats of India, females produce vocalizations that differ structurally (both temporally and spectrally) from males’ calls [31]. Females call spontaneously, even in the absence of males in breeding aggregations, and engage in agonistic interactions with other females. Although the authors did not explicitly define these vocalizations, they suggested female calls’ signal location and reproductive status and may also play a role in female–female competition when males are scarce.

Another example of sexual dimorphism in advertisement calls is observed in the concave-eared torrent frog (*Odorrana tormota*). In this species, females produce loud vocalizations at ultrasonic frequencies, while males exhibit a rich vocal repertoire with various call types [18,71–73]. This is perhaps the most extensively studied species regarding vocal behaviour in female frogs. In captivity, females call steadily throughout the night, and playback experiments and behavioural assays showed that female calls elicit positive phonotaxis from males. Studies conducted in captive and semi-natural conditions suggested that females can call spontaneously, attracting multiple males seeking mating opportunities. However, research on the function of these calls has been limited to female–male interactions, leaving the question of how females respond to the calls of other females unanswered. Despite researchers referring to these vocalizations as courtship calls, we propose that the female calls in *O. tormota* serve as advertisement calls.

The guardian frog of Borneo (*Limnonectes palavanensis*) is perhaps the best studied species concerning female–female acoustic interactions. Females vocalize spontaneously and more frequently than males, forming lek-like aggregations around a calling male. Males may respond to the female call with a low-amplitude courtship call [74]. Playback experiments show that females resume calling in response to female calls and increase their calling rate when both female and male calls are presented (J Goyes Vallejos, 2014, unpublished data). These findings suggest that the vocal behaviour of female *L. palavanensis* is analogous to the typical calling patterns of males of other species: calling spontaneously, more frequently, and eliciting responses from male and female conspecifics.

Current evidence suggests that the species described in this section produces advertisement calls, but testing their ‘dual’ function requires experiments with both males and females as intended receivers. This approach is critical to elucidate the functions of both female and male calls, since the function of advertisement calls in intrasexual interactions is often assumed but rarely tested.

## 5. Challenges in female calling behaviour research

### (a) Can you hear me?

Most reports describe female calls as short and low intensity, making them difficult to detect (e.g. [17,19,20]). This difference in call amplitude is often attributed to the absence of vocal sacs in females, though vocal sacs are not required for call production (e.g. [30]). Yet, there is some truth to these claims. Female calls tend to be softer, exhibit lower sound pressure levels and are harder to hear (e.g. [35,69,74]). Still, female calls with amplitudes similar to those of males are not uncommon, particularly in species with monomorphic calls (e.g. [23,75]).

Other factors hindering our ability to detect female calls in the field are the extremely male-biased operational sex ratios in breeding aggregations. Female calls may be drowned out by the overwhelming cacophony of frog choruses. Environmental noise further masks female calls, as seen in *Crossodactylus schmidti*, where extensive field observations yielded only seven analysable recordings of female courtship calls, likely due to the loud torrent streams where they breed (EM Santana, S Iop, MB dos Santos, VM Caldart, 2017-18, unpublished data).

Despite the difficulty in detecting female calls, the comprehensive list of all known frog species presented in this review emphasizes the prevalence of this behaviour across the anuran clade (electronic supplementary material, file S2). We encourage researchers to use this work as a reference resource for integrative studies of acoustic communication in anurans.

### (b) Mechanisms of sound production

At the anatomical level, males rely on their larynx to produce acoustic signals, but both sexes possess larynxes that primarily function in respiration [76]. Our understanding of the larynx’s role in female call production is still in its infancy. While males generally have larger larynxes [22,40,76,77], exceptions exist. In *Odorrana tormota* and *Staurois guttatus*, females have larynxes up to twice the size of males’ [40,78]. *Odorrana tormota* females produce multi-harmonic vocalizations extending into the ultrasonic range [60], while *S. guttatus* females generate complex, frequency-modulated calls with up to 35 notes—far more elaborate than the males’ simple two-note calls [40]. These examples challenge the notion that female calls are inherently ‘simple’ and ‘short’. However, despite their larger larynxes and distinct vocalizations, female calls in both species remain lower in intensity than male calls. This highlights an unexplored aspect regarding the mechanics of females’ call production and the functional implications of anatomical differences in anurans.

In contrast, the neuroendocrine mechanisms underlying anuran call production have been studied since the early 1990s, with the neuropeptide arginine vasotocin (AVT) identified as a key modulator of vocal behaviour. Further research revealed sexual dimorphism in these pathways and that AVT stimulates male calling behaviour [37,38,79–82]. Additional studies found that AVT can also trigger female calling, as demonstrated in *Hyla cinerea* females implanted with testosterone and treated with AVT, despite their typical lack of female calls [37]. This finding confirmed that females can call, even if they do not call in nature. However, despite this well-established framework, the effects of AVT on female frogs naturally producing vocalizations remain untested [38,80]. Some studies indicate that AVT inhibits release calls in female *A. catesbeiana* and *L. pipiens* [81,82], but its role in advertisement or courtship calls is currently unknown. The protocols and foundational knowledge exist, yet the proximate processes underlying female calling behaviour continue to be understudied.

### (c) The lack of functional tests

Various functions have been proposed to explain female calling behaviour in frogs. Some suggest that female calling aids in locating mates in challenging environments, such as habitats with high background noise or where individuals are widely dispersed [38,56,60]. This process may involve either males approaching females or vice versa, or female calls may elicit immediate phonotactic responses from males [72].

Traditionally, the function of frog vocalizations has been assessed through a combination of behavioural experiments, manipulations, field observations and playback experiments [83,84], among which playback experiments (i.e. phonotaxis) are the most used method. These experiments have been widely used to investigate the function of male frog calls, particularly in the context of mate attraction. Females are presented with recorded or synthetic male calls, and their preferences are inferred based on movement towards specific speakers.

There is a noticeable bias towards mate choice playback experiments that focus on males’ calls. Our initial database queries revealed this bias, returning significantly more articles on mating choice and calling interactions than on actual descriptions of female calls when using the keywords ‘female’ and ‘call’. Furthermore, playback experiments presenting male signals to other males are less common than those testing male calls with females, but they are still far more frequent than studies using female calls as stimuli for either males or females. In turn, playback experiments testing male responses to female calls exist for only a few species (e.g.[35,53,60,72,85,86]). Playback experiments using female calls as stimuli for other females or both sexes are even rarer, documented only in the Iberian midwife toad (*Alytes cisternasii*) [54] and the smooth guardian frog of Borneo (*Limnonectes palavanensis*) [87].

Some researchers have explored the function of female calls using behavioural manipulations. These include placing individuals together in an enclosure to observe interactions (e.g. [21,88]) or presenting an intruder, typically a male, to assess responses (e.g. [40,57]). However, important aspects such as the influence of the female reproductive state (e.g. gravid versus non-gravid) and female–female interactions remain largely unexamined. Finally, distress and release calls, which are often elicited through direct researcher manipulation, have rarely been investigated beyond simple descriptions.

Despite the increasing number of frog species that exhibit female calls, we know virtually nothing about inter- and intrasexual interactions from the female perspective. To advance the field, it is critical to shift our focus from predominantly studying male calls and begin conducting playback experiments that present female calls to both males and females.

## 6. Advancing bioacoustics research in anurans: the female perspective

### (a) The ‘male bias’

Reports of female calls have increased in recent years; however, confirming that females call in a species is inherently easier than demonstrating its absence, as this may result from a lack of detection. Because of this and the intrinsic acoustic properties of female calls (see §5a), their study remains underrepresented in the scientific literature and sound repositories.

Another significant barrier is the inconsistent and unclear reporting practices of female calling behaviour. Records of female calls are often buried within the main text of a publication, making them difficult to locate through automated searches. For example, sporadic descriptions of calling females exist in field guides, unpublished theses or books documenting anuran fauna focused on specific geographic regions (e.g. [23,30]). Yet they frequently lack a dedicated section on vocalizations that explicitly states whether females call and in what context.

Most accounts of female calls remain purely observational, often lacking context or sufficient detail to classify them into a call type. In several of the reviewed documents, female call reports were simply noted without further elaboration, and sometimes, it was not clear which species they were referring to. While caution is needed when defining calls without sufficient data, researchers should strive to go beyond simple descriptions by incorporating behavioural and ecological contexts where calls are produced.

Another prevalent practice in anuran communication studies is the omission of the sex of the vocalizing individual, reinforcing the assumption that only males vocalize, a male bias also observed in other taxa (e.g. birds [89]). In frogs, this bias leads to the use of terms such as ‘advertisement call’ instead of ‘male advertisement call’, making it harder to identify instances of female calling in literature searches. The lack of standardized keywords for female vocalizations further limits discoverability. To improve documentation, we urge researchers to explicitly note female calling in abstracts and keywords (e.g. ‘female anuran call’). Reviewers and editors should also ensure that studies on acoustic communication specify the sex of the vocalizing individual, helping to correct these long-standing biases.

A related pitfall is the routine classification of all calling individuals as ‘male’ based solely on the observation of the call itself, without further verification (e.g. [90,91]). This issue extends to sound repositories where frog recordings are often labelled as ‘male’ as if calling behaviour automatically infers sex. This is particularly critical for species with monomorphic calls, where females produce calls that are indistinguishable from males. Consequently, previous descriptions of male-only calls may inadvertently include female calls. To improve data reliability, researchers should always verify the caller’s sex using multiple criteria, such as body size, checking for sexually dimorphic traits (e.g. vocal sacs), presence of eggs, and the behavioural context in which these calls are observed or recorded.

### (b) Absence of evidence is not evidence of absence

Improved reporting practices are critical for documenting female calling, including the need to document ‘negative data’—whether females of a given species have ever been observed vocalizing. Without such data, comparative analyses investigating the evolution of female calls are not possible. A study on *Acris crepitans* exemplifies proper reporting: researchers noted that females do not vocalize in a courtship, distress or release contexts and have found no evidence of such behaviours in the literature or in their work with multiple populations [76]. Another example comes from research on *Xenopus* frogs, for which Tobias and collaborators [21] experimentally tested for the occurrence of female release calls across 18 species. They concluded that female release calls are absent in 11 of these *Xenopus* species. Approaches like this are not only possible and necessary, but they should become standard practice in the future if we aim to develop a more integrative view of anuran communication.

### (c) Female call evolution

Some hypotheses have been proposed to explain the evolution of female calls. An early hypothesis suggested that anuran calls evolved from simple distress signals involved in predator avoidance [92–95]. However, conducting phylogenetic comparative analyses to study the evolution of male advertisement calls is not feasible due to their widespread occurrence in nearly all frog species [17]. Instead, female frogs seem poised for this type of study, as accurate records become increasingly available, laying the groundwork for testing hypotheses regarding the evolution of anuran vocalizations.

Studying species where female calling occurs alongside specific life-history traits could provide deeper insights into its evolution. One key area is the relationship between female calling and parental care. In *Alytes* species, males carry their eggs around their legs until they hatch [96], while females produce courtship calls and display aggression towards other females in captivity [97]. It has been suggested that ‘reciprocal calls’ (here defined as courtship calls) evolved in species with extensive male parental care [46]. The premise is that male parental care could lead to a female-biased operational sex ratio, promoting female–female competition for mates and/or male mate choice [46], driving the evolution of sexually selected signals in females [98]. However, this hypothesis lacks empirical support, as male parental care has evolved independently in several frog lineages without documented female calls [99].

Without empirical testing, it remains difficult to make broad generalizations about the evolutionary drivers of female calling behaviour. Comparative studies across anurans will be crucial for identifying the ecological and evolutionary factors that have shaped the evolution of the different female call types.

## 7. Future directions and research opportunities

Our understanding of female calling in frogs remains limited, particularly regarding its costs and benefits. Future research should examine the trade-offs of call production, including energetic investment, predation risk and fitness consequences. Bioacoustics analyses are also lacking. Comparative studies should assess the spectral and temporal properties of both female and male calls as a cohesive system, considering how environmental factors influence sound propagation and signal reception. Similarly, studies should explore how females adjust their calling behaviour in response to other individuals, including timing, spatial positioning and interactions with conspecifics. Identifying the social contexts of female calls—whether related to mate attraction, competition or other contexts—will improve our grasp of their specific functions.

Sexual selection remains another understudied area. It is unclear whether female calls influence mate attraction, whether males discriminate among calling females, or whether female–female competition occurs in species where females produce advertisement calls or aggressive calls. For example, in *Staurois guttatus*, females produce more elaborate aggressive calls than males [40]. These instances provide an opportunity to investigate the evolutionary pressures shaping complex female signals. Future research should also consider multimodal signalling from the female perspective. In *Odorrana tormota*, females use both calls and visual signals (‘blinking’) to communicate with males [18]. Investigating how female frogs integrate different signal types could clarify their role in sexual selection and communication.

Overall, the significant gaps in research regarding female anuran calls present numerous opportunities for future research. The historical focus on male calls reflects a broader bias within the scientific community. However, this trend is shifting, especially as more women researchers lead studies bringing the female role to the forefront [89,100–102]. We hope this review positively impacts the study of anuran communication and promotes a more inclusive approach that considers both sexes.

## Data Availability

Additional data are available from Dryad Digital Repository [103]. Supplementary material is available online [104].

## Dedication

J.G.V. dedicates this paper to the memory of Kentwood D. Wells (1948–2024). His early contributions, along with his *magnum opus*, were instrumental in our understanding of the complexities of social behaviour in frogs. It was a privilege to be his last graduate student, and through this work, I aspire to reflect even a fraction of his remarkable ability to present information in an engaging and concise manner.
